# Impact of the Hydrolysis and Methanolysis of Bidesmosidic *Chenopodium quinoa* Saponins on Their Hemolytic Activity

**DOI:** 10.3390/molecules27103211

**Published:** 2022-05-17

**Authors:** Philippe Savarino, Carolina Contino, Emmanuel Colson, Gustavo Cabrera-Barjas, Julien De Winter, Pascal Gerbaux

**Affiliations:** 1 Organic Synthesis and Mass Spectrometry Laboratory (S²MOs), University of Mons—UMONS, 23 Place du Parc, 7000 Mons, Belgium; philippe.savarino@umons.ac.be (P.S.); carolina.tiziana.c@gmail.com (C.C.); colson-emmanuel@outlook.be (E.C.); julien.dewinter@umons.ac.be (J.D.W.); 2 Unidad de Desarrollo Tecnológico (UDT), Universidad de Concepción, Av. Cordillera 2634, Parque Industrial Coronel, Concepción 4030000, Región del Bío Bío, Chile; g.cabrera@udt.cl

**Keywords:** saponins, mass spectrometry, structure-activity relationship, chemical modification, hemolytic activity

## Abstract

Saponins are specific metabolites abundantly present in plants and several marine animals. Their high cytotoxicity is associated with their membranolytic properties, i.e., their propensity to disrupt cell membranes upon incorporation. As such, saponins are highly attractive for numerous applications, provided the relation between their molecular structures and their biological activities is understood at the molecular level. In the present investigation, we focused on the bidesmosidic saponins extracted from the quinoa husk, whose saccharidic chains are appended on the aglycone via two different linkages, a glycosidic bond, and an ester function. The later position is sensitive to chemical modifications, such as hydrolysis and methanolysis. We prepared and characterized three sets of saponins using mass spectrometry: (i) bidesmosidic saponins directly extracted from the ground husk, (ii) monodesmosidic saponins with a carboxylic acid group, and (iii) monodesmosidic saponins with a methyl ester function. The impact of the structural modifications on the membranolytic activity of the saponins was assayed based on the determination of their hemolytic activity. The natural bidesmosidic saponins do not present any hemolytic activity even at the highest tested concentration (500 µg·mL^−1^). Hydrolyzed saponins already degrade erythrocytes at 20 µg·mL^−1^, whereas 100 µg·mL^−1^ of transesterified saponins is needed to induce detectable activity. The observation that monodesmosidic saponins, hydrolyzed or transesterified, are much more active against erythrocytes than the bidesmosidic ones confirms that bidesmosidic saponins are likely to be the dormant form of saponins in plants. Additionally, the observation that negatively charged saponins, i.e., the hydrolyzed ones, are more hemolytic than the neutral ones could be related to the red blood cell membrane structure.

## 1. Introduction

**Figure 1:**
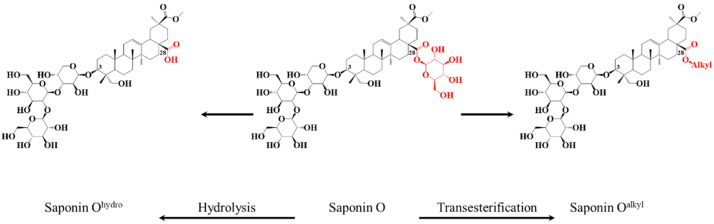
Specific chemical modifications of the bidesmosidic saponins extracted from *Chenopodium quinoa* husk: (i) hydrolysis of Saponin O to Saponin O^hydro^, and (ii) transesterification of Saponin O to Saponin O^alkyl^.

For many years, molecules of natural origin have been a research topic of interest, due to their structural diversity and complexity, but also for their biological properties, which can be of major industrial interest if correctly understood and mastered. Within these numerous classes of biomolecules, specific metabolites, such as alkaloids, flavonoids, and saponins, are a hot research topic due to their specific interactions with living organisms [1,2,3]. Among these specific metabolites, saponins have been demonstrated to fulfill defensive roles, intervene in inter- and intra-species communications, or even play a role in reproduction processes [4,5,6,7,8,9,10]. These molecules are abundantly present in plants [11], and are also present in a diversity of marine animals, like sponges and echinoderms [12,13]. Saponins present a specific structural identity consisting of the association between an apolar aglycone and one or more (linear or branched) glycans. Monodesmosidic and bidesmosidic saponins are respectively constituted by a single or two saccharidic chains anchored on a single aglycone [14]. Diverse specific chemical functions, such as sulfate groups [15], free carboxylic acid (−COOH) [16], esterified acetic, tiglic or angelic acids [17,18], and many others [19,20], are also often present on saponins and modulate the saponin biological activities [21,22,23,24,25]. The membranolytic activity of saponins, i.e., their propensity to disrupt the cell membrane upon interaction with membrane sterols, represents one of the most interesting properties for pharmaceutical applications [26,27,28,29,30]. Computational chemistry studies have recently made it possible to visualize the saponin/membrane interaction at the molecular level and represent a promising tool for identifying structural moieties responsible for the activity [31,32,33,34] on the way to the establishment of the Structure–Activity Relationship (SAR) [26]. From an experimental point of view, selective and specific modification of chemical functions using organic chemistry methods represents an elegant method for evaluating their contribution to membranolytic activity [35,36]. As a typical example, there is a general agreement that bidesmosidic saponins are less cytotoxic than monodesmosidic ones [28]. In this context, we recently successfully converted the bidesmosidic saponins extracted from the husk of the Chilean *Chenopodium quinoa* Willd. (1798) into their monodesmosidic ones [37,38,39] upon specific microwave-assisted hydrolysis of the ester bond at C28 (see Figure 1). The cytotoxicity of the hydrolyzed saponins was shown to be significantly enhanced with regards to the natural bidesmosidic saponins [40]. More recently, we investigated the importance of the sulfate function as a cytotoxicity vector for saponins contained in the viscera of the Malagasy sea cucumber *Holothuria scabra* [41]. Under microwave activation, the sulfated saponins were quantitatively converted into their desulfated counterparts, and the comparison of the hemolytic activities (HA) of both sets of saponins revealed that the sulfate group was mandatory for the membranolytic activity [41]. Several similar studies have been reported in the literature, i.e., esterification of tea saponins [42], amide group derivatization of β-hederin [43], and selective modification of the glycan or the aglycone of chlorogenin-type saponins [44]. We strongly believe that these combined efforts will contribute to the understanding of the cytotoxicity of saponins at the molecular level.

In the present study, we re-examined the cytotoxicity of the saponins found in the husk of the quinoa seeds [45]. Our motivation comes from the fact that, even if quinoa seeds are known for their very high nutritional value (rich in protein (~20%) and antioxidant compounds) [45] and their ease of plant cultivation in almost any conditions [37,38], the husk, which represents approximatively 10% of the weight of the seed, is currently discarded due to the large concentration of saponins. Then, it can be used as a source of value-added products with applications in pharmacy, agriculture, and foods, which is in line with the Circular Economy policies promoted by the EU, provided the biological properties of the natural molecules and their easily accessible derivatives can be fully identified.

In our previous study [40], we demonstrated that monodesmosidic saponins, such as **Saponin O^hydro^** produced from **Saponin O**, shown in Figure 1, are more cytotoxic than the natural bidesmosidic saponins. The cytotoxicity of saponins is often associated with their amphiphilic nature making their association with cell membrane favorable [28]. We thus suspected that the neutralization of the carboxylate group present at C28 on the hydrolyzed saponins should enhance their cytotoxicity. Here, we report on the impact of the transesterification, using potassium methanolate in methanol, of the bidesmosidic saponins extracted from quinoa husk on their cytotoxicity. To achieve this objective, we compare the hemolytic activities of three different fully characterized samples: (i) natural saponins extracted from the quinoa husk, (ii) C-28 hydrolyzed saponins, and (iii) C-28 transesterified saponins. All the samples are qualitatively and quantitatively characterized using mass spectrometry methods, in light of the support of literature data [37,39,40].

## 2. Results and Discussion

### 2.1. Saponin Identification and Quantification in the Natural Extract (NE)

**Figure 2:**
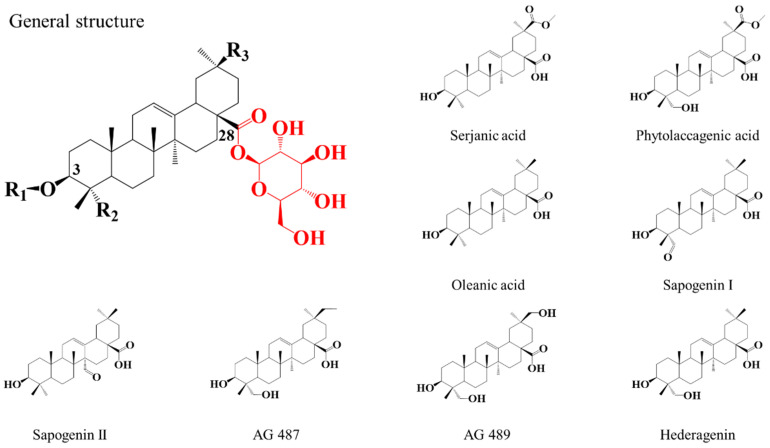
General structure of the bidesmosidic saponins extracted from the quinoa husk. R_1_ corresponds to the C3-attached glycan as detailed in Table 1. R_2_ and R_3_ functions are specific to the aglycone moiety as shown in the presented aglycones. The C28-glucose is highlighted in red since this residue will be involved in the chemical modifications targeted, i.e., hydrolysis and methanolysis.

The characterization of the saponins contained in the NE is achieved using the mass spectrometry (MS) protocol developed in our laboratory [46], combining MALDI-MS, accurate mass measurements (HRMS) and LC-MS (MS) experiments. The saponin identification is based on reference studies by Madl et al. [37], Kuljanabhagavad et al. [39] and Colson et al. [40]. The quinoa saponins are bidesmosidic (C3 and C28) triterpenoidic saponins and have the particularity to possess a single glucose residue on C28 [37,39], see Figure 2. Their structure differences arise from (i) the number and the nature (glucose—Glu, galactose—Gal, arabinose—Ara, xylose—Xyl, glucuronic acid—GlcA) of the saccharide units composing the C3-attached glycan, and from (ii) the structure of the triterpene aglycone (oleanic acid—OA, hederagenin—Hed, AG489, AG 487, serjanic acid—SA, phytolaccagenic acid—PA, sapogenin I—SGI, sapogenin II—SGII) [37,39], see also Figure 2.

The saponin NE is obtained by methanol extraction of the ground husks, followed by successive liquid/liquid extractions, as described in the “Materials and Methods” section [47]. The yield of extraction is 310.06 mg per 20 g of ground husk, i.e., 15.5 mg·g^−1^.

**Figure 3:**
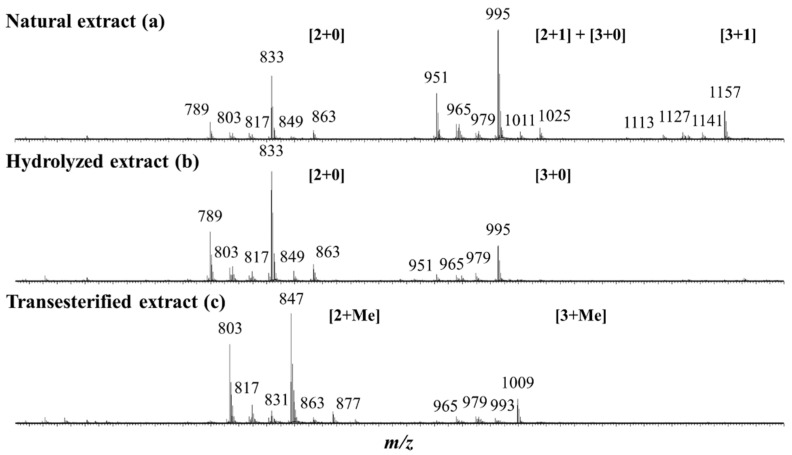
MALDI mass spectrometry analysis of three different saponin extracts: (**a**) the natural extract (NE); (**b**) the microwave-assisted alkaline hydrolysis (pH 10—150 °C—5 min) reaction products; and (**c**) the transesterification using MeOK (MeOH_anh_—N_2_—60 °C—60 min) reaction products. The saponin ion assignment was performed using the [x + y] symbolism, where x and y stand for the number of monosaccharide residues at C3 and C28, respectively. [x + Me] indicates that the C-28 glucose residue has been substituted by a methoxy group. Please note that the monodesmosidic [2 + 0] and [3 + 0] saponin ions detected in the NE (**a**) are produced during the MALDI processes from the corresponding bidesmosidic saponins (see text).

Keeping in mind the literature data [37,39,40] revealing that quinoa husk saponins are bidesmosidic three- and four-sugar saponins, the NE is first qualitatively and quantitatively analyzed by mass spectrometry and all the data are presented in Table 1. The MALDI-MS(+) mass spectrum presents three groups of *m*/*z* signals, see Figure 3a. These signals are ascribed to sodium-cationization saponins, [M + Na]^+^ [37,39,40]. The presence of monodesmosidic and bidesmosidic saponins must be considered a priori, and these saponins will be identified as [x + y], where x and y stand for the number of monosaccharide residues at C3 and C28, respectively. Please note that monodesmosidic saponins are not expected in the NE based on literature data [37,39,40], but we previously showed that monodesmosidic saponin ions may be generated during the MALDI-MS analysis [40]. The first group of saponin ions (*m*/*z* 1113–1157) corresponds to four-saccharide saponin ions, the second group (*m*/*z* 951–1025) corresponds to three-saccharide saponin ions, and the third group (*m*/*z* 789–863) corresponds to unexpected two-saccharide saponin ions. In the MALDI-MS spectrum presented in Figure 3a, we therefore assign to the *m*/*z* 951–1025 saponin ions the [2 + 1] and [3 + 0] topologies, whereas the *m*/*z* 1113–1157 ions are purely [3 + 1] ions and the *m*/*z* 789–863 ions are [2 + 0] fragment ions, as shown in the literature [40] and confirmed below using LC-MS analysis. Let us again emphasize that, when a saponin extract is exposed to mass spectrometry analysis, depending on the selected ionization method, either Electrospray or MALDI, fragment ions may be generated. This is the case here, as demonstrated in [40], for the bidesmosidic saponins extracted from the quinoa husk that suffer an ester bond dissociation under MALDI conditions [40].

Table 1 presents the elemental compositions of all the MALDI-observed [M + Na]^+^ ions determined based on HRMS measurements. Twenty different elemental compositions were detected, and are all gathered in Table 1. Please note that, in Figure 3a, only the most intense signals are assigned for readability reasons. LC-MS and LC-MSMS analyses are further mandatory to (i) confirm that the detected ions are saponin ions, (ii) discriminate between monodesmosidic and bidesmosidic saponin ions, (iii) identify potential isomers, and (iv) determine the glycan sequence and the aglycone nature using collision-induced dissociation (CID) experiments. Our LC analysis confirmed that the NE exclusively contains bidesmosidic saponins with 12 different elemental compositions and no isomers (see Figures S1 and S2) and that the two-sugar saponin ions detected between *m*/*z* 789 and 863 in the MALDI spectrum in Figure 3a are [2 + 0] fragment ions produced from the [2 + 1] saponins during the MALDI ionization. Additionally, some of the three-sugar saponin ions (*m*/*z* 951–1025) detected in Figure 3a are [3 + 0] fragment ions from the [3 + 1] bidesmosidic saponins. This also confirms that saponin quantification using MALDI is not relevant. The LC-MSMS spectra of the most abundant saponins ions, allowing the determination of the aglycone and saccharide sequence, are shown in Figures S3–S5. Please note that, upon LC-MS (MS), the saponins are mainly detected as [M + H]^+^ ions and that the CID spectra of all the quinoa saponin [M + H]^+^ ions have already been presented in our previous study [40].

**Figure 4:**
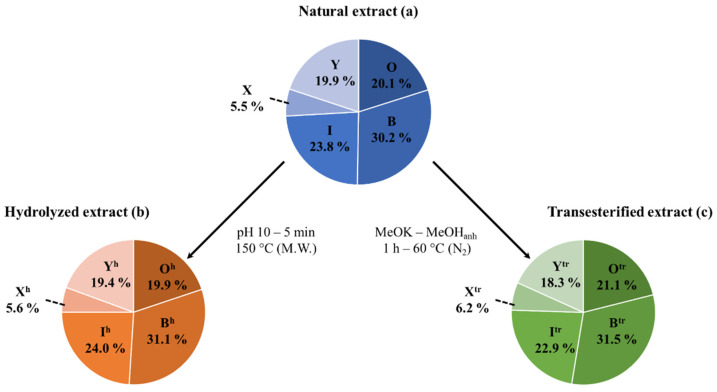
Mass spectrometry qualitative and quantitative analysis of the (**a**) natural, (**b**) hydrolyzed, and (**c**) transesterified saponin extracts: the saponin relative proportions (%) correspond to the molar proportions as determined by LC-MS signal integration. Please note that the relative proportions of Saponins X and Saponins Y correspond to the sum of the proportions of saponins G, 32 and 61 and the proportions of saponins N, 4, Q, H, 19 and F, respectively. M.W. stands for microwave activation.

Extracted Ion Current (EIC) chromatograms (see Figures S1 and S2) are used for the determination of the molar proportions of all the different bidesmosidic saponins detected in the NE by integrating the corresponding ion signals. Among the 12 saponin ions detected by LC-MS, *m*/*z* 1135 (Saponin O), *m*/*z* 973 (Saponin B), and *m*/*z* 929 (Saponin I) are the most abundant saponins in the NE, with molar proportions around 20, 30 and 24%, respectively, see Table 1. We will pool all the other minor saponins (26% molar proportion) together according to their compositions, e.g., 3-sugar vs. 4-sugar saponins. Saponins G, 32 and 61 will accordingly be gathered as saponins X (~6% molar ratio) and saponins N, 4, Q, H, 19 and F as saponins Y (~20%). These data are presented as a sector diagram in Figure 4a for further comparison. Using Hederacoside C, a commercially available saponin extracted from *Hedera helix*, as an internal standard, the saponin %-weights in the NE were determined for the 12 elemental compositions in Table 1. The three major saponins, namely Saponin O, Saponin B and Saponin I, represent respectively ~20%, ~30% and ~22% in weight of the dried extract, while the pooled saponins X and saponins Y, represent ~6% and ~18%, leading to a saponin weight percentage of 95.91% in the extract, i.e., 95.91 mg of saponins per 100 mg of dry extract. The saponin %-weight in the extract was further converted in the saponin mass fraction (mg·g^−1^) in the ground husk, using the extraction gravimetric yield previously determined at 15.5 mg of extract per g of ground husk. The three major saponins are present at ~3 (Saponin O), ~4.5 (Saponin B), and ~3.5 (Saponin I) mg per g of husk powder, while the minor saponins were estimated to be present around ~0.9 (Saponins X), and ~2.8 (Saponins Y) mg·g^−1^ of husk powder, see Table 1.

### 2.2. Selective Hydrolysis and Transesterification of the Quinoa Husk Bidesmosidic Saponins at C28

**Figure 5:**
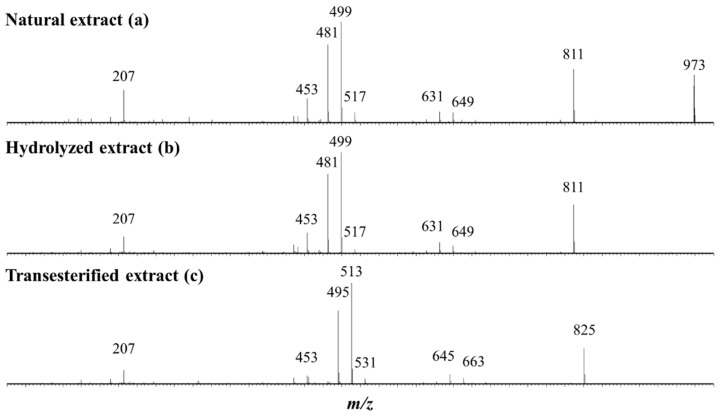
LC-MSMS analysis of the (**a**) natural extract, (**b**) hydrolyzed extract, and (**c**) transesterified extract. Collision-induced dissociation (CID) mass spectra of the (**a**) *m*/*z* 973, (**b**) *m*/*z* 811 and (**c**) *m*/*z* 825 precursor ions, respectively, corresponding to the [M + H]^+^ ions of (**a**) Saponin B, (**b**) hydrolyzed Saponin B, and (**c**) transesterified Saponin B.

The bidesmosidic saponins of the NE, see Figure 1, are first hydrolyzed under microwave activation to produce the monodesmosidic saponins bearing a carboxylate group at C28, generating the so-called hydrolyzed extract (HE). This reaction was previously developed in our laboratory [40], but, since we are conducting a comparative study, the intrinsic variability of the saponin natural extract makes it necessary to qualitatively and quantitatively characterize the hydrolysis products. Figure 3b presents the MALDI mass spectrum recorded after microwave-assisted hydrolysis and immediately confirms the success of the hydrolysis, since the bidesmosidic [3 + 1] saponin ions can no longer be detected. Further, HRMS measurements and LC-MS and LC-MSMS experiments confirm that the saponin ions detected in the *m*/*z* 951–995 mass range correspond to monodesmosidic [3 + 0] saponins and that the *m*/*z* 789–963 ions are [2 + 0] saponins. The [3 + 1] and [2 + 1] saponins can no longer be detected, testifying to the success of the hydrolysis. As a typical example, we compare in Figure 5a,b the LC-MSMS mass spectra of the [M + H]^+^ ions from (a) Saponin B (*m*/*z* 973) and hydrolyzed Saponin B (*m*/*z* 811). Upon collisional activation (see also Figure S4), the *m*/*z* 973 Saponin B ions first expel the C-28 glucose residue to generate the fragment ions detected at *m*/*z* 811 that ultimately decompose to yield the aglycone ions detected at *m*/*z* 499. These *m*/*z* 811 ions also correspond, from the hydrolyzed extract, to the [M + H]^+^ ions of the C-28 hydrolyzed Saponin B. The CID spectrum of these *m*/*z* 811 ions is presented in Figure 5b, and a comparison of Figure 5a,b unambiguously confirms that the hydrolysis reaction is specific at the C-28 position, since all the detected fragment ions detected below *m*/*z* 811 are identical.

The data are presented in Table 2, as well as in Figure 4, for quantitative analysis. The comparison of the sector diagrams built for the NE and the HE also confirms that the hydrolysis reaction is specific to the C28 ester bond, since the relative proportions between the different saponins are conserved upon hydrolysis. In other words, saponins O (20.1%), B (30.2%), I (23.8%), X (5.5%) and Y (19.9%) are quantitatively (~100% yield) converted into saponins O^h^ (19.9%), B^h^ (31.1%), I^h^ (24.0%), X^h^ (5.6%), and Y^h^ (19.4%), see Figure 4.

As shown in Figure 1, the third set of saponins targeted for our comparative study is constituted by C28-esterified saponins. Two strategies can be borrowed from organic chemistry corresponding to the direct esterification of the hydrolyzed saponins and the transesterification of the bidesmosidic saponins. All attempts, see Figure S6, to esterify the hydrolyzed saponins at the C28 position failed, and the C28 carboxylic acid/carboxylate moiety was systematically recovered after reaction [48,49]. We further tested several protocols, see Figure S7, for the transesterification of the natural bidesmosidic saponins [50]. As shown in Figure 1, potassium methanolate (MeOK, 1 M), in anhydrous methanol (MeOH_anh_) for 1 h at 60 °C, efficiently produces the C28-methylated saponins, yielding so-called Transesterified Extract (TE). Indeed, as shown in Figure 3c, the signals attributed to the bidesmosidic saponin ions can no longer be detected after MeOK treatment. The [M + Na]^+^ ions are now detected at 148 u (mass unit) lower than the bidesmosidic saponin ions. This mass difference, confirmed upon HRMS measurements (see Table 3), corresponds to the formal substitution of a glucose residue by a methoxy group. Globally, the MALDI mass spectrum features two groups of ions that correspond to the [3 + Me] and [2 + Me] saponins, respectively, in the *m*/*z* 965–1009 and *m*/*z* 803–877 mass ranges. LC-MS and LC-MSMS analyses confirm that the transesterification of the bidesmosidic saponins is quantitative and selective, since all the bidesmosidic saponins constituting the NE are now detected as their C28-methylated counterparts in the TE. Again, as a typical example, the LC-MSMS spectra of the [M + H]^+^ ions of Saponin B (*m*/*z* 973), hydrolyzed Saponin B (*m*/*z* 811), and transesterified Saponin B (*m*/*z* 825) are compared in Figure 5. It is significant to observe that all the fragment ions from the [M + H]^+^ ions of transesterified Saponin B are shifted to 14 u mass higher than the fragment ions from the [M + H]^+^ ions of hydrolyzed Saponin B. This strongly supports our conclusion that the structural modification under the MeOK treatment is specific to the C-28 ester function.

Finally, the molar proportions of the different saponins remain largely unaffected upon transesterification, as shown in the graphical comparison in Figure 4, where saponins O (20.1%), B (30.2%), I (23.8%), X (5.5%) and Y (19.9%) are quantitatively converted into saponins O^tr^ (21.1%), B^tr^ (31.5%), I^tr^ (22.9%), X^tr^ (6.2%), and Y^tr^ (18.3%), see Figure 4.

**Figure 6:**
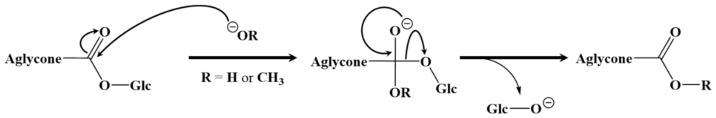
Mechanistic proposal for the hydrolysis (HO^−^) and transesterification (CH_3_O^−^) reactions undergone by the bidesmosidic saponins extracted from the quinoa husk: addition–elimination mechanism specifically occurring at the ester function.

The hydrolyzed and transesterification reactions performed on the bidesmosidic [3 + 1] and [2 + 1] saponins extracted from the quinoa husk were demonstrated to specifically occur for the C-28 ester function. We propose, in accordance with basic organic chemistry, that both processes involve a nucleophilic addition of HO^−^ (basic hydrolysis) or CH_3_O^−^ (transesterification) at the carbon atom of the C-28 ester function, followed by an elimination of the C-28 glucose as the leaving group, according to the general mechanism presented in Figure 6.

### 2.3. Hemolytic Activity (HA) Modulation

The membranolytic properties of NE, HE and TE are compared by determining their hemolytic activities (HA) as a standard method [29,35,47,51,52,53,54]. HA is evaluated by determining the evolution of the hemoglobin release in solution when a suspension of red blood cells is exposed to increasing concentrations of the tested molecules. The hemoglobin release is measured by determining the solution absorbance at 540 nm [55]. We recently proposed the use of a referent saponin solution to make it possible to compare results from different groups [40,41], and we selected the highly hemolytic saponins extracted from *Aesculus hippocastanum* [56]. The HA are therefore expressed as a percentage of the activity of the standard solution (see Material and Methods).

**Figure 7:**
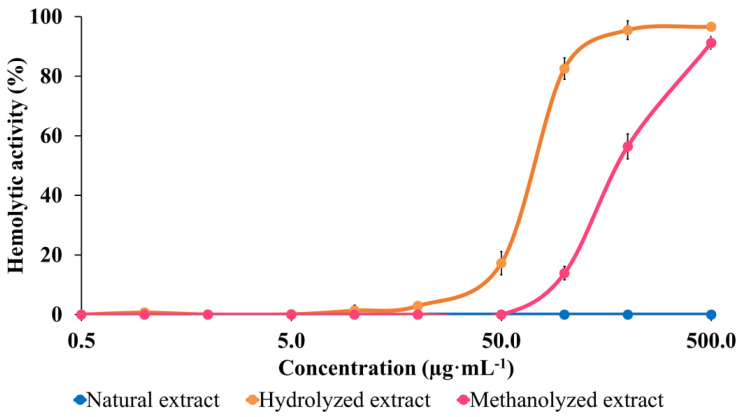
Cytotoxicity evaluation of the three saponin extracts: hemolytic activity comparison between the natural bidesmosidic saponins, the hydrolyzed saponins, and the transesterified saponins. Experiments were performed in triplicate, using a 2% erythrocytes suspension from bovine blood. Hemolytic activities are expressed in % of the activity of the referent, a 500 µg·mL^−1^ solution of *A. hippocastanum* saponins.

The comparison of the HA of the three extracts, see Figure 7, undoubtedly demonstrates the impact of chemical modifications on the HA. The data first confirm that (i) the bidesmosidic saponins present in the NE do not present any membranolytic activity against the red blood cells in the used concentration range, say up to 500 µg·mL^−1^; and (ii) that monodesmosidic saponins present in the hydrolyzed extract are already active at a concentration around 20 µg·mL^−1^. The bidesmosidic saponins are strongly activated upon transesterification, since a HA is detected as being above 50 µg·mL^−1^, see Figure 7. This also reveals that the hydrolyzed negatively charged saponins are more membranolytic than the transesterified ones, which is at odds with our prediction on the basis of their presumed relative amphiphilicities.

The red blood cell membrane, being rich in *N*-acetyl-neuraminic acids, is globally negatively charged [57,58]. This permanent global negative charge is mandatory for preventing red blood cells from aggregating and also for creating a high concentration in positive ions all around the red blood cells [57,58]. The greater activity of the hydrolyzed saponins that exhibit a net negative charge may be linked to this accumulation of positive charges around the red blood cells. We recently showed that the desulfation of the negatively charged sulfated saponins extracted from *Holothuria scabra* generates neutral saponins whose HA can no longer be detected [41].

## 3. Materials and Methods

### 3.1. Chemicals

Technical-grade methanol, *n*-hexane, chloroform, dichloromethane and isobutanol, HPLC grade water, formic acid, acetonitrile, and methanol were provided by CHEM-LAB NV (Somme-Leuze, Belgium). 2,5-dihydroxybenzoic acid (DHB), N,N-dimethylaniline (DMA), Hederacoside C and potassium methanolate were purchased from Sigma-Aldrich (Diegem, Belgium). Phosphoric acid, borax, sodium hydroxide and hydrochloric acid were provided by VWR Chemicals (Leuven, Belgium), while thionyl chloride, *p*-toluenesulfonic acid, sulfuric acid and Dowex^TM^ resin were purchased from Thermo Scientific (Merelbeke, Belgium).

### 3.2. Extraction

Mature achene integuments were obtained from pooled samples (Spring 2020) from the Quinoa Breeding Program from Instituto Nacional dé Investigación Agria (INIA) Chile. Seeds were then subjected to physical shearing and kernels were discarded. The obtained husk powder (particle diameter < 1 mm) was sent to Belgium and kept away from light. The husk powder was placed under stirring overnight in methanol. The solution was then centrifuged at 4500× *g* for ten minutes (Sigma 2-16P, Sigma, Osterode am Harz, Germany). The supernatant was then collected, and the extract diluted with water to reach a volume ration of 70/30 (methanol/water). This methanolic extract was partitioned (*v*/*v*) with *n*-hexane, chloroform, and dichloromethane to remove apolar compounds. The third aqueous phase is recovered and evaporated under vacuum using a rotary evaporator (IKA RV 10, IKA, Staufen, Germany) in a water bath (80 rpm, 50 °C) and the residue is brought to a volume of 25 mL in order to carry out a fourth liquid/liquid extraction (*v*/*v*) with HPLC isobutanol to recover the saponins in the organic phase. This phase is then washed twice with Milli-Q water to purify the extract from the residual salts and impurities. The organic phase, containing saponins, is evaporated under vacuum to obtain a purified powder.

### 3.3. Microwave-Assisted Alkaline Hydrolysis

The hydrolysis protocol was adapted from our previous study [40]. *C. quinoa* NE (3 mg) is solubilized in 3 mL of a buffer solution (pH 10:50 mL of borax 0.025 mol·L^−1^ added to 18.3 mL of NaOH 0.1 mol·L-1, brought to 100 mL with Milli-Q water). The solution is heated at 150 °C for 5 min using a microwave device (Biotage, Initiator Classic, Biotage Sweden, Uppsala, Sweden) and cooled to room temperature. The pH is brought to 7 using HCl 0.1 mol·L^−1^ and a liquid/liquid extraction is performed (*v*/*v*) with isobutanol. The organic phase is washed twice with Milli-Q water and evaporated under vacuum to obtain the saponins of HE in a powder (57% yield, 97% conversion).

### 3.4. Methanolysis

The transesterification protocol was adapted from Chung et al. [50]. *C. quinoa* NE (100 mg) is placed overnight in a vial at 50 °C to remove residual water. The vial is then placed in a graphite bath (60 °C, under N_2_) and 15 mL of CH_3_OK (1 mol·L^−1^) in anhydrous methanol are added (stirring, 60 min). The solution is directly evaporated under vacuum and the dry extract is brought to a volume of 15 mL with isobutanol before undergoing two liquid/liquid extractions (*v*/*v*) with Milli-Q water to desalt the phase. The butanol phase is again evaporated to dryness under vacuum to recover the TE saponins as a powder (60% yield, 95% conversion).

### 3.5. Mass Spectrometry (MS) Analyses

The MS analyses are carried out using Matrix-assisted Laser Desorption/Ionization (MALDI), performed on a Water QToF Premier mass spectrometer (Waters, Manchester, UK) in the positive ionization mode. The matrix consists of a mixture of dihydroxybenzoic acid (DHB, 25 mg) and N,N-dimethylaniline (DMA, 6 µL, 99.9%) in 250 µL of Milli-Q water/acetonitrile (*v*/*v*). A matrix solution droplet (1 µL) is placed on a stainless-steel plate and air-dried. An amount of 1 µL of the sample solution (1 mg of dried extract in 1 mL of HPLC grade methanol) is then spotted on the top of the matrix crystal and air-dried. The plate is introduced into the MALDI-ToF mass spectrometer. The MALDI source is composed of an Nd-YAG laser with a maximum energy of 104.1 µJ, transferred to the sample in a 2.2 ns pulse (200 Hz). For spectral recording, the quadrupole (rf-only mode) si configured to let the ions pass between *m*/*z* 250 and 2000. All the ions are then mass-analyzed using the ToF analyzer (1 s integration time). Mass analyses are performed with the ToF analyzer in reflectron mode, at a FWHM resolution around 10,000. Accurate mass measurements (HRMS) are performed using MALDI-MS(+) with PEG 600-1500 as the external standard (lock mass).

Liquid chromatography analyses are performed with a Waters Acquity UPLC H-Class (Waters, Manchester, UK) composed of a vacuum degasser, a quaternary pump and an autosampler, coupled to a Waters Synapt G2-S*i* mass spectrometer (Waters, Manchester, UK). A non-polar column (Acquity UPLC BEH C18; 2.1 × 50 mm; 1.7 µm; Waters) is used at 40 °C. For these analyses, 0.1 mg of saponin extract is dissolved in 1 mL of a Milli-Q water/acetonitrile solution (85/15). A volume of 5 µL is injected into the column. The gradient is optimized for the compounds in this study and follows a flow rate of 250 µL·min^−1^ of Milli-Q water (with 0.1% formic acid (HCOOH), eluent A) and acetonitrile (CH_3_CN, eluent B). The mobile phase consists of an elution gradient starting with 85% of eluent A and 15% of eluent B, reaching 60% of eluent A and 40% eluent B at 6 min, and maintained for 3 min. The ratio is then modified to reach 5% eluent A and 95% eluent B at 11 min, maintained for 1 min and, finally, brought back to 85% eluent A and 15% eluent B at 13 min. This ratio is maintained until the end of the chromatographic run (15 min). Electrospray ionization (ESI) in positive ionization mode is used for the saponin ion production with typical conditions as follows: capillary voltage 3.1 kV, cone voltage 40 kV, source offset 80 V, source temperature of 120 °C and desolvation temperature of 300 °C (dry nitrogen flow rate 500 L·h^−1^), for a mass range (quadrupole in rf-only mode) between *m*/*z* 50 and 2000 (1 s integration time). For the LC-MSMS experiments, precursor ions are mass-selected by the quadrupole and collided against argon (Ar) in the Trap cell of the TriWave^R^ device, and the kinetic energy of the laboratory frame (E_lab_) is selected to afford intense enough product ion signals. The fragment ions are mass-measured with the ToF analyzer.

The relative quantification of saponins within the natural extract is achieved by adding a known quantity (0.1 mg·mL^−1^) of commercially available Hederacoside C (Sigma-Aldrich—Product n° 97151—M-Clarity^TM^ Program MQ100), a pure saponin from *Hedera helix*, as internal standard in a solution of saponin extract at a given concentration, typically 0.1 mg·mL^−1^. The spiked solution is analyzed using LC-MS (5 µL injection volume) using the experimental conditions described above. For each saponin molecule, including Hederacoside C, the corresponding LC-MS ion signals—including all the isotopic compositions—are integrated using the integration algorithm, available under MassLynx^TM^ 4.1 Software (Waters, Manchester, UK). The global ion counts are then used to estimate the concentration of each saponin, relatively to Hederacoside C signal integration. The %-weights in extract (see Table 1) correspond to the mass percentages of saponins with a given elemental composition within the saponin extract. Please note that the sum of the %-weight does not reach 100%, making it possible to estimate the saponin content within the extract at 95.9%. The mass fractions in viscera expressed in mg·g^−1^ (see Table 1) are further calculated by using the global yield of extraction determined at 15.5 mg of extract per g of ground husk.

### 3.6. Hemolytic Activity Experiments

To measure the hemolytic activity, reflecting the membranolytic activity, bovine blood (stored with 0.11 M sodium citrate) was collected immediately after the death of the animal at the Abattoirs de Ath (22 Chemin des Peupliers, 7800 Ath, Belgium) on 10 April 2021. The bovine blood was then washed using a phosphate buffered saline (PBS) solution. This solution was prepared by dissolving 8 g of sodium chloride (NaCl), 1.45 g of sodium hydrogen phosphate dihydrate (Na_2_HPO_4_·2H_2_O), 0.2 g of potassium chloride (KCl) and 0.2 g potassium dihydrogen phosphate (KH_2_PO_4_) in 800 mL of Milli-Q water. The pH of the solution was adjusted to 7.4 and the solution was brought to a volume of 1 L using Milli-Q water. In a 50 mL Falcon, 10 mL of citrated bovine blood were added to 40 mL of PBS solution. The Falcons were then centrifuged for fifteen minutes at 10,000 g and the pellet was preserved. The washing was repeated until a clear and colorless supernatant was obtained. The supernatant was discarded and 2 mL from the pellets was diluted with 98 mL of PBS, to obtain a 2% (*v*/*v*) erythrocyte suspension. At the same time, various solutions containing the extract of saponins at different concentrations were prepared. The latter were placed in the presence of the 2% erythrocyte suspension in triplicate and incubated for one hour at 20 °C, with continuous shaking (500 rpm) before being centrifuged again at 10,000× *g* for ten minutes. The supernatant of each sample was then collected to measure the absorbance of the solution (540 nm) [59]. In our assay, we systematically used a 500 µg·mL^−1^ solution of saponins extracted from *Aesculus hippocastanum* seeds as a reference solution, since the corresponding escins are highly membranolytic [56]. The HA of the tested saponin solutions were then calculated using the following equation:

(1) $$\mathrm{HA}\;\left(\%\right)=\frac{\left({\mathrm{Abs}}_{\mathrm{sample}}-{\mathrm{Abs}}_{\mathrm{blank}}\right)}{\left({\mathrm{Abs}}_{\mathrm{ref}}-{\mathrm{Abs}}_{\mathrm{blank}}\right)}*100$$

where Abs_sample_, Abs_blank_, and Abs_ref_, respectively, correspond to the absorbance (540 nm) of the tested erythrocytes/saponins solutions, of the erythrocyte solution and of the erythrocyte/referent saponin solution.

## 4. Conclusions

The elucidation of the relation between the biological activity of a family of molecules and their molecular structures make it possible to explore the role of specific chemical moieties present on active molecules on their biological activities.

In the present investigation, we focused on the bidesmosidic saponins extracted from the *C. quinoa* husk, whose saccharidic chains were appended on the aglycone via two different linkages, a glycosidic bond in C3 and an ester function in C28. The C28 position was therefore sensitive to chemical modifications, such as hydrolysis and transesterification. We thus prepared three sets of saponins: (i) bidesmosidic saponins directly extracted and purified from the ground husk (NE—Natural Extract), (ii) monodesmosidic saponins with a carboxylic acid group in C28 (HE—Hydrolyzed Extract), and (iii) monodesmosidic saponins with a methyl ester function in C28 (TE—Transesterified Extract). The HE and TE saponins were respectively prepared by microwave-assisted alkaline hydrolysis and transesterification with potassium methanolate in anhydrous methanol under inert atmosphere from the NE saponins. Mass spectrometry experiments demonstrate that the hydrolysis and the transesterification are both highly specific to the C28 ester function and quantitative (~100% conversion). The impact of the structural modifications on the membranolytic activity of the natural saponins was then assayed on the basis of hemolytic activity measurement. The natural bidesmosidic saponins were confirmed to have no activity against erythrocytes even at the highest tested concentration (500 µg·mL^−1^). The hydrolyzed saponins start to be active against red blood cells already at 20 µg·mL^−1^, whereas 50 µg·mL^−1^ of the transesterified saponins are necessary for inducing a detectable hemoglobin release from the destroyed red blood cells. Globally, the observation that monodesmosidic saponins, hydrolyzed or transesterified, are much more active against erythrocytes than the bidesmosidic ones confirms that bidesmosidic saponins are likely to be the dormant form of saponins in plants [60]. On the other hand, negatively charged saponins, i.e., the hydrolyzed ones, being more hemolytic than the neutral ones, could be associated with the high concentrations of positive charged ions in the vicinity of the negatively charged red blood cell membranes. We detected a similar effect with sulfated saponins (SO_4_^−^) that were shown to be no longer hemolytic upon desulfation [41]. These results pointing to the role of charged groups of saponins on their biological activity should be addressed in the future for targeting specific applications.

## Figures and Tables

**Table 1:** *Chenopodium quinoa* husk extract: data collected by MS-based experiments. The compositions and mass error measurements (Δ) were determined by MALDI-HRMS. ^a^ The saponins were identified based on liquid chromatography (LC-MS) and collision-induced dissociation experiments (LC-MSMS). ^b^ The saponin ions detected between *m*/*z* 789 and 863 are [2 + 0] fragment ions generated during the MALDI ionization from the [2 + 1] saponins. The %-weights in extracts and the mass fractions (mg·g^−1^ of *Chenopodium quinoa* husk powder) were determined based on the LC ion signal intensity ratios, with Hederacoside C as an internal standard, and using the gravimetric extraction yield (15.5 mg·g^−1^). The molar proportions (%) were determined based on LC ion signal relative integration. See the “Materials and Methods” section for the details of all the quantitative analysis.

Saponins	Elemental Compositions (M)	*m*/*z* (Δ ppm) [M + Na]^+^	Aglycone	3-*O* Glycan (R_1_)	%-Weights in Extract (%)	Mass Fractions in Husk (mg·g^−1^)	Retention Times (min)	Molar Proportions (%)
O ^a^	C_54_H_86_O_25_	1157.5356 (1.6)	PA	Glu-Glu-Ara-	20.01 ± 0.11	3.101	**5.34**	**20.11 ± 0.13**
G ^a^	C_54_H_86_O_24_	1141.5407 (0.6)	SA	Glu-Glu-Ara-	0.29 ± 0.03	0.045	6.31	0.34 ± 0.05
32 ^a^	C_53_H_84_O_24_	1127.5250 (3.2)	PA	Glu-Ara-Ara-	3.46 ± 0.02	0.536	4.89	3.62 ± 0.03
61 ^a^	C_53_H_86_O_23_	1113.5458 (1.2)	Hed	Glu-Glu-Ara-	1.98 ± 0.06	0.307	6.51	2.03 ± 0.07
N ^a^	C_49_H_78_O_21_	1025.4933 (3.2)	PA	Glu-Gal-	3.89 ± 0.10	0.603	4.77	4.00 ± 0.13
4 ^a^	C_48_H_76_O_21_	1011.4777 (1.1)	AG533	Glu-Ara-	0.07 ± 0.03	0.011	5.43	0.09 ± 0.03
B ^a^	C_48_H_76_O_20_	995.4828 (0.1)	PA	Glu-Ara-	29.50 ± 0.09	4.572	**5.39**	**30.16 ± 0.09**
Q ^a^	C_48_H_78_O_19_	981.5035 (3.3)	Hed	Glu-Gal-	1.87 ± 0.10	0.290	5.92	1.97 ± 0.11
H ^a^	C_48_H_76_O_19_	979.4878 (4.1)	SA	Glu-Ara-	0.29 ± 0.09	0.045	6.31	0.33 ± 0.12
19 ^a^	C_47_H_76_O_19_	967.4878 (1.6)	AG489	Glu-Ara-	5.11 ± 0.07	0.792	3.72	5.83 ± 0.10
F ^a^	C_47_H_74_O_19_	965.4722 (1.4)	Hed	Xyl-GlcA-	7.01 ± 0.10	1.086	4.92	7.69 ± 0.05
I ^a^	C_47_H_76_O_18_	951.4929 (2.0)	Hed	Glu-Ara-	22.43 ± 0.11	3.476	**6.58**	**23.83 ± 0.14**
/ ^b^	C_43_H_68_O_16_	863.4405 (0.6)	/	/	/	/	/	/
/ ^b^	C_42_H_66_O_16_	849.4249 (1.9)	/	/	/	/	/	/
/ ^b^	C_42_H_66_O_15_	833.4299 (0.1)	/	/	/	/	/	/
/ ^b^	C_42_H_68_O_14_	819.4507 (2.1)	/	/	/	/	/	/
/ ^b^	C_42_H_66_O_14_	817.4350 (0.5)	/	/	/	/	/	/
/ ^b^	C_41_H_66_O_14_	805.4350 (5.0)	/	/	/	/	/	/
/ ^b^	C_41_H_64_O_14_	803.4194 (0.7)	/	/	/	/	/	/
/ ^b^	C_41_H_66_O_13_	789.4401 (0.1)	/	/	/	/	/	/

**Table 2:** Microwave-assisted alkaline hydrolysis (pH 10—150 °C—5 min) of *Chenopodium quinoa* husk saponin extract: the elemental compositions of saponin ions are determined by MALDI-HRMS and the molar proportions (%) of the saponin ion are estimated based on the LC-MS signal relative integration.

Saponins	Elemental Compositions (M)	*m*/*z* [M + Na]^+^	Mass Errors (Δ ppm)	Retention Times (min)	Molar Proportions (%)
O^h^	C_48_H_76_O_20_	995.4828	2.8	**7.02**	**20.06 ± 0.08**
G^h^	C_48_H_76_O_19_	979.4878	0.8	8.71	0.23 ± 0.01
32^h^	C_47_H_74_O_19_	965.4746	2.3	6.51	3.35 ± 0.03
61^h^	C_47_H_76_O_18_	951.4929	3.0	6.71	1.98 ± 0.03
N^h^	C_43_H_68_O_16_	863.4405	0.6	6.78	4.43 ± 0.04
4^h^	C_42_H_66_O_16_	849.4249	1.9	7.42	0.05 ± 0.01
B^h^	C_42_H_66_O_15_	833.4299	0.1	**7.23**	**30.31 ± 0.02**
Q^h^	C_42_H_68_O_14_	819.4507	2.1	8.76	1.95 ± 0.03
H^h^	C_42_H_66_O_14_	817.4350	0.5	8.81	0.35 ± 0.03
19^h^	C_41_H_66_O_14_	805.4350	5.0	5.28	5.75 ± 0.04
F^h^	C_41_H_64_O_14_	803.4194	0.7	6.67	7.95 ± 0.03
I^h^	C_41_H_66_O_13_	789.4401	0.1	**10.14**	**23.59 ± 0.10**

**Table 3:** Transesterification (MeOK 1 M—MeOH_anh_—N_2_—60 °C—60 min) of *Chenopodium quinoa* husk bidesmosidic saponins: the elemental compositions of saponin ions are determined by MALDI-HRMS, and the molar proportions (%) of all the saponin ions are estimated based on the LC-MS signal integration.

Saponin	Elemental Composition (M)	*m*/*z* [M + Na]^+^	Mass Error (Δ ppm)	Retention Time (min)	Composition Molar Proportion (%)
O^tr^	C_49_H_78_O_20_	1009.4984	1.6	**9.47**	**20.09 ± 0.06**
G^tr^	C_49_H_78_O_19_	993.5035	3.5	11.45	0.21 ± 0.01
32^tr^	C_48_H_76_O_19_	979.4878	0.2	8.74	3.34 ± 0.05
61^tr^	C_48_H_78_O_18_	965.5086	1.5	11.81	1.91 ± 0.03
N^tr^	C_44_H_70_O_16_	877.4198	0.2	9.89	4.46 ± 0.11
4^tr^	C_43_H_68_O_16_	863.4405	2.2	9.33	0.06 ± 0.01
B^tr^	C_43_H_68_O_15_	847.4456	1.7	**10.72**	**30.26 ± 0.11**
Q^tr^	C_43_H_70_O_14_	833.4663	3.1	7.00	1.88 ± 0.03
H^tr^	C_43_H_68_O_14_	831.4507	1.7	7.86	0.32 ± 0.04
19^tr^	C_42_H_68_O_14_	819.4507	4.8	7.34	5.70 ± 0.06
F^tr^	C_42_H_66_O_14_	817.4350	5.0	9.57	7.94 ± 0.03
I^tr^	C_42_H_68_O_13_	803.4558	1.0	**11.99**	**23.83 ± 0.10**

## Data Availability

The data presented in this study are available on request from the corresponding author.

## Supplementary Materials

The following supporting information can be downloaded at: https://www.mdpi.com/article/10.3390/molecules27103211/s1, Figure S1: LC-MS analysis of the natural saponin extract: EIC (Extracted Ion Current Chromatogram) of *m*/*z* 1135, *m*/*z* 1119, *m*/*z* 1105, and *m*/*z* 1091, respectively corresponding to [M + H]^+^ ions of extracted bidesmosidic [3 + 1] saponins from *Chenopodium quinoa* husk; Figure S2: LC-MS analysis of the natural saponin extract: EIC (Extracted Ion Current Chromatogram) of *m*/*z* 1003, *m*/*z* 989, *m*/*z* 973, *m*/*z* 959, *m*/*z* 957, *m*/*z* 945, *m*/*z* 943, and *m*/*z* 921, respectively corresponding to [M + H]^+^ ions of extracted bidesmosidic [2 + 1] saponins from *Chenopodium quinoa* husk; Figure S3: LC-MSMS(+) analysis of *Chenopodium quinoa* husk saponin extract: CID spectrum (10 eV) recorded for the *m*/*z* 1135 precursor ions [M + H]^+^ at 5.34 min retention time (Saponin O); Figure S4: LC-MSMS(+) analysis of *Chenopodium quinoa* husk saponin extract: CID spectrum (10 eV) recorded for the *m*/*z* 973 precursor ions [M + H]^+^ at 5.39 min retention time (Saponin B); Figure S5: LC-MSMS(+) analysis of *Chenopodium quinoa* husk saponin extract: CID spectrum (10 eV) recorded for the *m*/*z* 929 precursor ions [M + H]^+^ at 6.58 min retention time (Saponin I); Figure S6: Direct esterification of monodesmosidic saponins (from the hydrolyzed extract—HE): unsuccessful attempts. Invariably the starting material is recovered after reaction; Figure S7: Methanolysis of the bidesmosidic saponins (from the natural extract—NE). All attempts under neutral/acidic conditions failed and only the transesterification using MeOK in anhydrous methanol under inert atmosphere afforded the expected C28-methylated saponins.

## Sample Availability

Samples of the compounds are available from the authors upon request.
