# Supplementary material for: Impact of the Hydrolysis and Methanolysis of Bidesmosidic Chenopodium quinoa Saponins on Their Hemolytic Activity
Source: Molecules. 2022 May 17;27(10):3211. doi: 10.3390/molecules27103211 (PMC9144749; doi:10.3390/molecules27103211)
Supplement: Supplementary file 1 [file molecules-27-03211-s001.zip › molecules-1627225-supplementary.pdf]

molecules

Impact of the Hydrolysis and Methanolysis of Bidesmosidic *Chenopodium quinoa* Saponins on Their Hemolytic Activity

Philippe Savarino [1], Carolina Contino [1], Emmanuel Colson [1], Gustavo Cabrera-Barjas [2], Julien De Winter [1] and Pascal Gerbaux [1,*]

[1] Organic Synthesis and Mass Spectrometry Laboratory (S²MOs), University of Mons, 23 Place du Parc, 7000 Mons, Belgium
[2] Unidad de Desarrollo Tecnológico (UDT), Universidad de Concepción, Av. Cordillera 2634, Parque Industrial Coronel, Coronel P.O. Box 4051 mail 3, Región del BíoBío, Chile

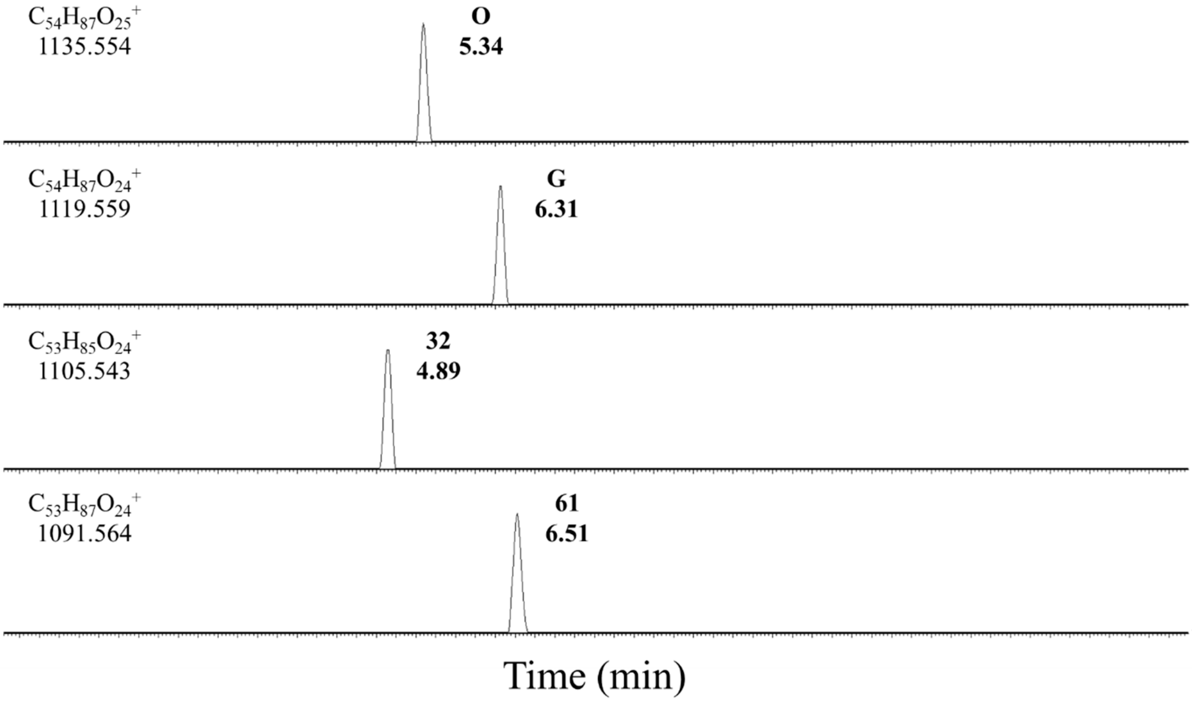

Figure S1. LC-MS analysis of the natural saponin extract: EIC (Extracted Ion Current Chromatogram) of *m/z* 1135, *m/z* 1119, *m/z* 1105, and *m/z* 1091, respectively corresponding to $[M+H]^+$ ions of extracted bidesmosidic [3+1] saponins from *Chenopodium quinoa* husk.

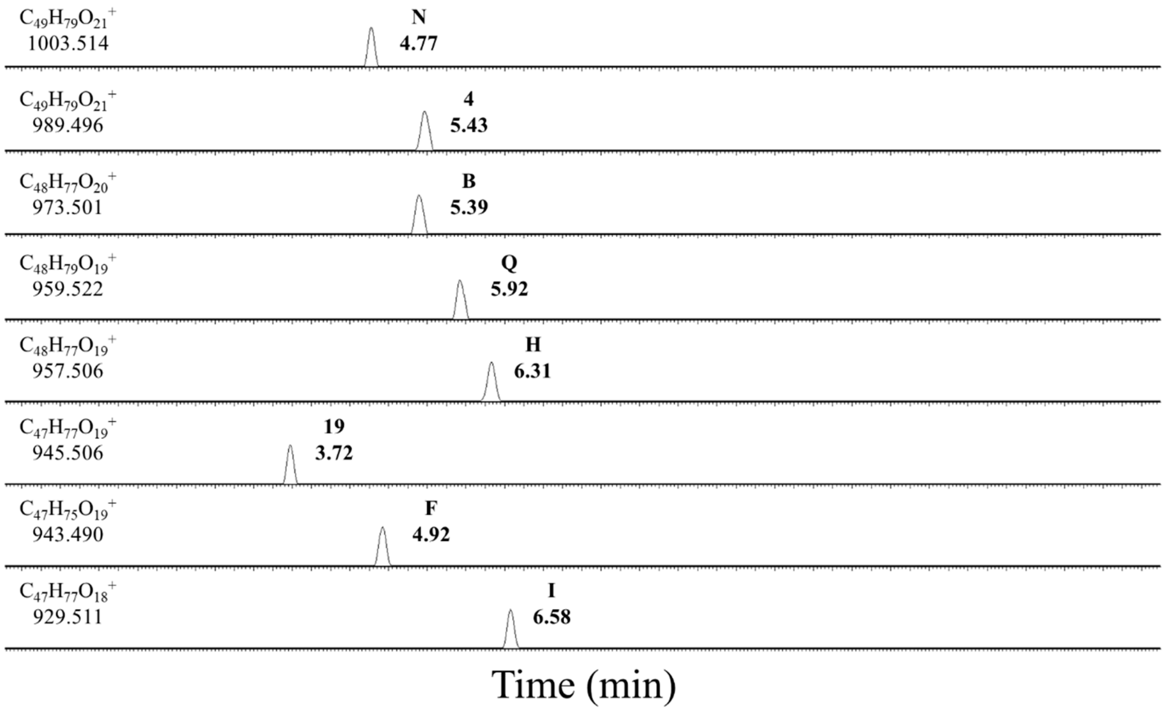

Figure S2. LC-MS analysis of the natural saponin extract: EIC (Extracted Ion Current Chromatogram) of *m/z* 1003, *m/z* 989, *m/z* 973, *m/z* 959, *m/z* 957, *m/z* 945, *m/z* 943, and *m/z* 921, respectively corresponding to [M+H]+ ions of extracted bidesmosidic [2+1] saponins from *Chenopodium quinoa* husk.

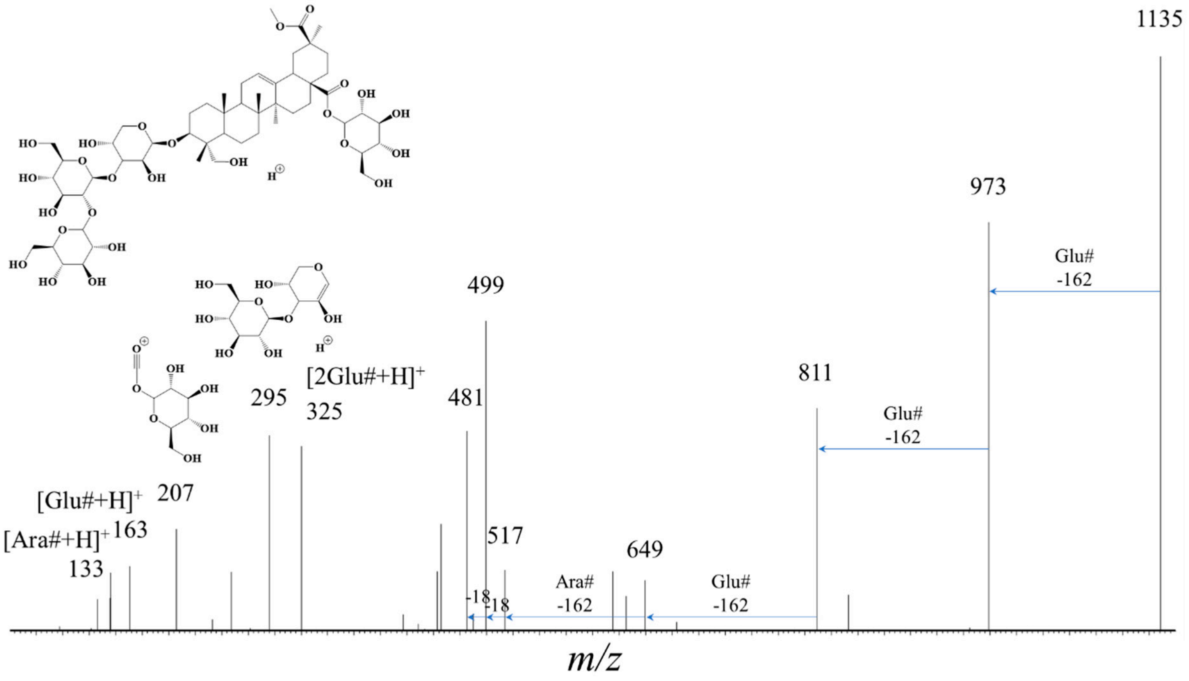

Figure S3. LC-MSMS(+) analysis of *Chenopodium quinoa* husk saponin extract: CID spectrum (10 eV) recorded for the *m/z* 1135 precursor ions [M+H]+ at 5.34 min retention time (Saponin O).

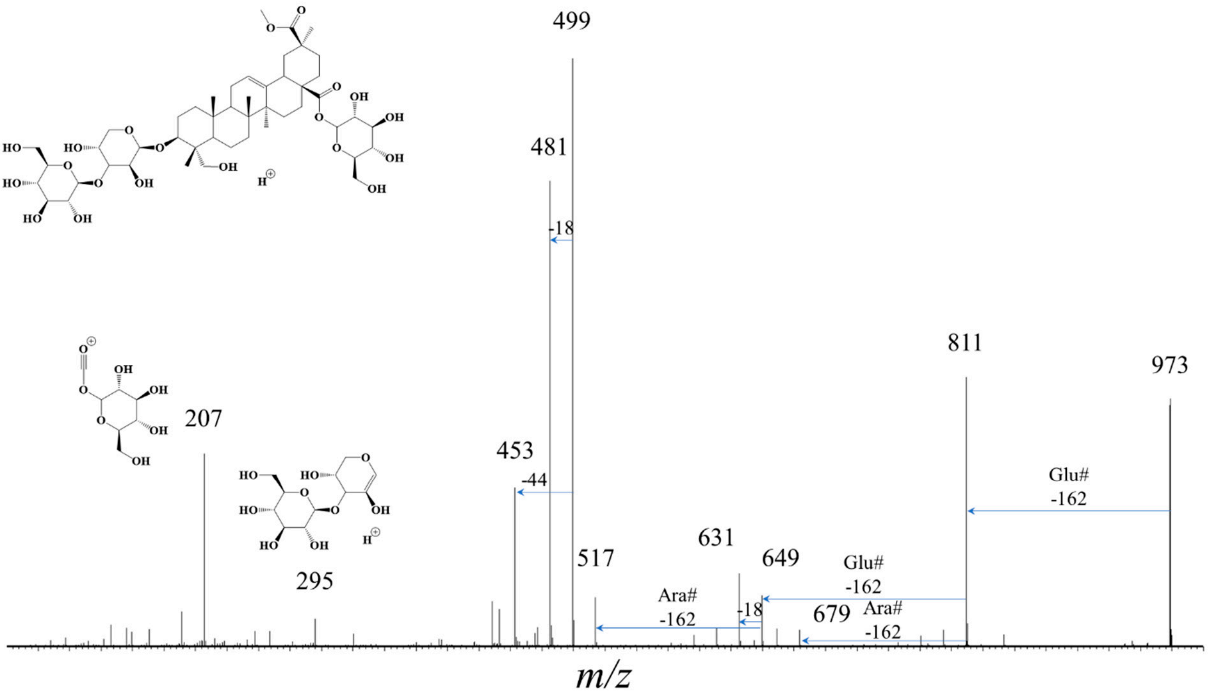

Figure S4. LC-MSMS(+) analysis of *Chenopodium quinoa* husk saponin extract: CID spectrum (10 eV) recorded for the *m*/*z* 973 precursor ions $[M+H]^+$ at 5.39 min retention time (Saponin B).

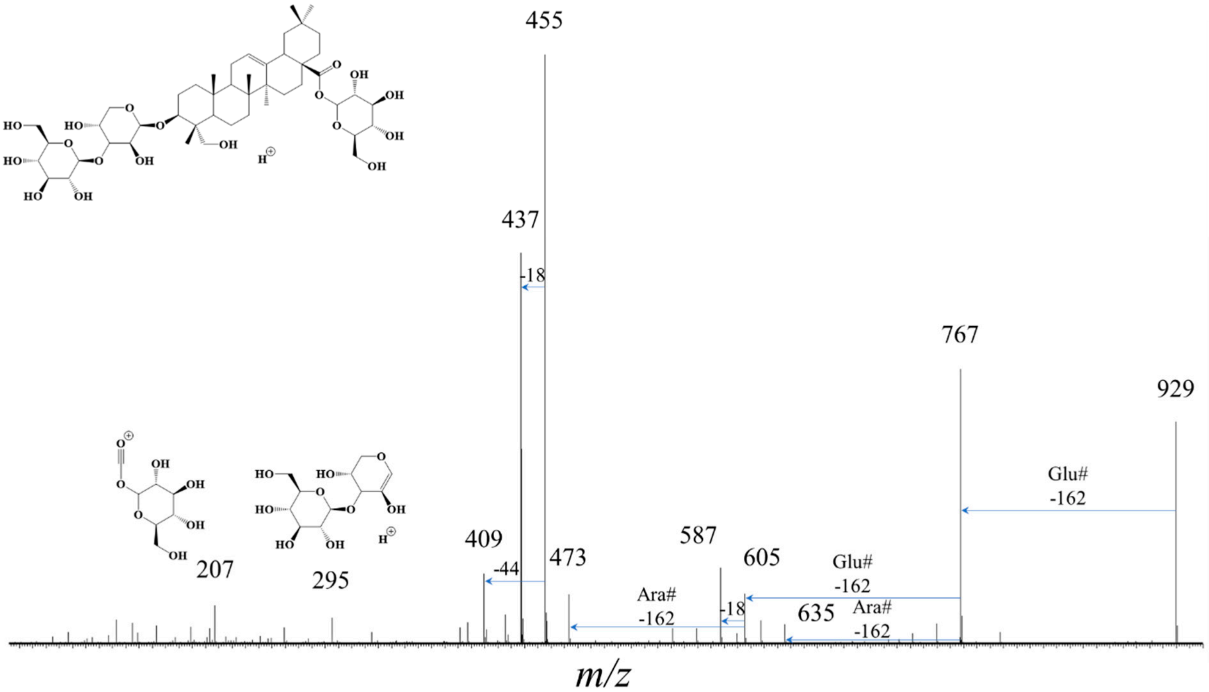

Figure S5. LC-MSMS(+) analysis of *Chenopodium quinoa* husk saponin extract: CID spectrum (10 eV) recorded for the *m*/*z* 929 precursor ions $[M+H]^+$ at 6.58 min retention time (Saponin I).

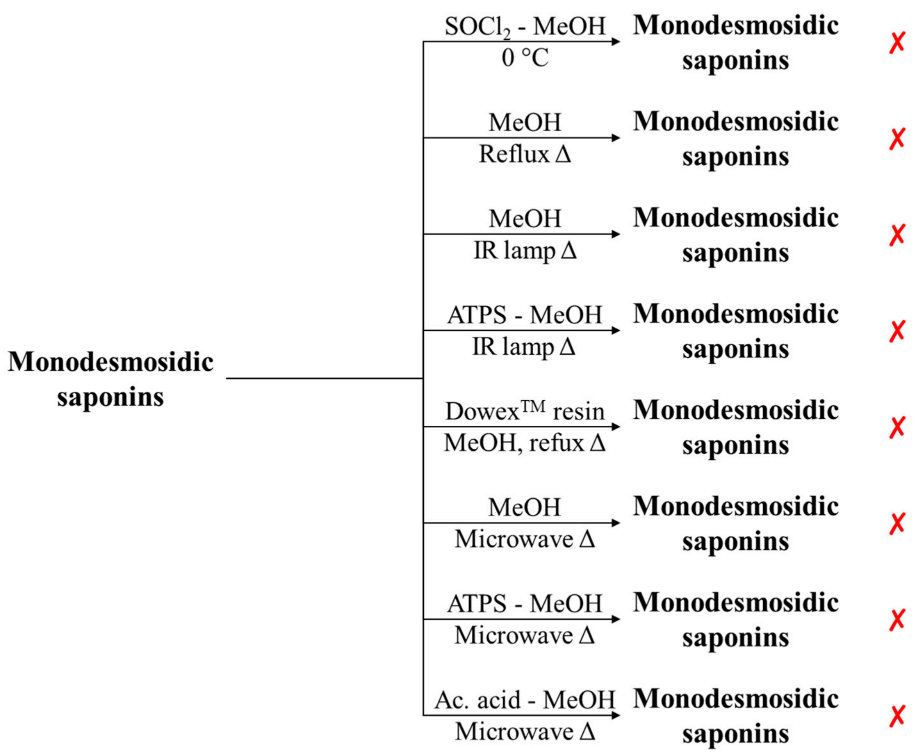

Figure S6. Direct esterification of monodesmosidic saponins (from the hydrolyzed extract - HE): unsuccessful attempts. Invariably the starting material is recovered after reaction.

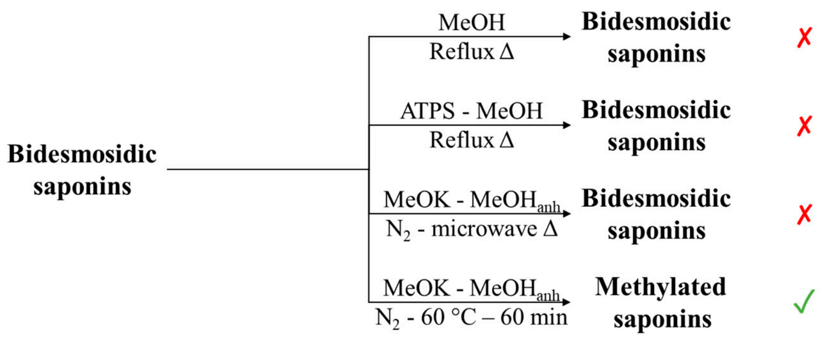

Figure S7. Methanolysis of the bidesmosidic saponins (from the natural extract - NE). All attempts under neutral/acidic conditions failed and only the transesterification using MeOK in anhydrous methanol under inert atmosphere afforded the expected C28-methylated saponins.
